# Integrative analysis of postharvest chilling injury in cherry tomato fruit reveals contrapuntal spatio-temporal responses to ripening and cold stress

**DOI:** 10.1038/s41598-019-38877-0

**Published:** 2019-02-26

**Authors:** Karin Albornoz, Marita I. Cantwell, Lu Zhang, Diane M. Beckles

**Affiliations:** 1 Department of Plant Sciences, University of California, One Shields Avenue, Davis, CA 95616 United States; 2 Department of Biological and Agricultural Engineering, University of California, One Shields Avenue, Davis, CA 95616 United States; 3 School of Biological Sciences, The University of Hong Kong, Pok Fu Lam Road, Hong Kong, China

## Abstract

Postharvest chilling injury (PCI) reduces fruit quality and shelf-life in tomato (*Solanum lycopersicum* L.). PCI has been traditionally studied in the pericarp, however its development is likely heterogeneous in different fruit tissues. To gain insight into PCI’s spatio-temporal development, we used postharvest biomarkers e.g. respiration and ethylene rates, ion leakage etc., to confirm the occurrence of PCI, and compared these data with molecular (gene expression), biophysical (MRI data) and biochemical parameters (Malondialdehyde (MDA) and starch content) from the pericarp or columella. Tissues were stored at control (12.5 °C) or PCI-inducing temperatures (2.5 or 5 °C) followed by rewarming at 20 °C. MRI and ion leakage revealed that cold irreversibly impairs ripening-associated membrane liquefaction; MRI also showed that the internal and external fruit tissues responded differently to cold. MDA and especially starch contents, were affected by chilling in a tissue-specific manner. The expression of the six genes studied: *ACO1* and *ACS2* (ripening), *CBF1* (cold response*), DHN*, *AOX1a* and *LoxB* (stress-related) showed non-overlapping temporal and spatially-specific responses. Overall, the data highlighted the interconnectedness of fruit cold response and ripening, and showed how cold stress reconfigures the latter. They further underscored that multidimensional spatial and temporal biological studies are needed to develop effective solutions to PCI.

## Introduction

Refrigeration is the most effective tool to prevent postharvest losses^1^ of fruits and vegetables, however, its utilization is limited in cold-sensitive commodities, which typically originate from tropical and subtropical regions^2–4^. The term ‘postharvest chilling injury’ or ‘PCI’ is used to describe the group of symptoms and physiological alterations that compromise quality and promote spoilage when sensitive commodities are stored at temperatures between 0 and 15 °C^3,5^. PCI contributes to postharvest horticultural crop loss, which is unsustainable given the need to produce more food for a burgeoning global population using fewer natural resources^6,7^. Chilling injury has been studied in numerous species for more than 200 years, yet our understanding of the progress of its early stages and underlying causes at the molecular level is still incomplete, hindering the development of long-term solutions to this problem.

Tomato (*Solanum lycopersicum* L.) is the second most important vegetable crop, ranking number one in terms of gross production value in the world^8^. It is a key source of antioxidants for humans^9,10^ and is also a model organism for the study of fleshy-fruited species^11–13^. Tomato suffers from PCI: storage at 0–12 °C followed by returning the fruit to room temperature can lead to a range of symptoms, varying from mild e.g. lack of flavor and poor texture, to severe i.e. development of surface lesions, discoloration, accelerated softening, failure of fruit to ripen and higher susceptibility to postharvest decay^14^.

Numerous studies in tomato fruit, including those on PCI, have traditionally focused on only one tissue, the pericarp^15,16^. Since PCI is a complex phenomenon, the lack of robust, practical solutions could be a consequence of the use of fragmentary approaches when analyzing the progression of the disorder. The pericarp and the central tissue, the columella, account for most of the fruit fresh mass in round tomatoes^17,18^, however, in cherry tomato the pericarp is comparatively thinner and the placenta and locular tissue are significantly larger^19^, and contribute to most of what is eaten. Chilling can affect internal tissues^20,21^ and there is abundant evidence describing the differential development of physiological and biochemical processes in the different tomato fruit fractions^15,16,22–28^.

It is also known that PCI targets processes occurring across different biological levels and time frames^14^. The precise order of events triggered by PCI is unknown, but one of the primary events is the production of reactive oxygen species (ROS)^14,29–31^. If cold exposure is mild or limited, cellular homeostasis and fruit quality will be maintained through the activation of alternative oxidases, and protective proteins such as dehydrins, in part, by the regulation of upstream factors of the cold response pathways such as C-Binding Repeat transcription Factors (CBFs)^32,33^. Beyond this threshold, or during rewarming of previously cold-stored fruit, progressive loss of selective membrane permeability due to lipid peroxidation may occur. This in turn, can lead to secondary metabolic and physiological dysfunction such as the leakage of water, solutes and metabolites, ROS accumulation, bursts in ethylene and respiratory rates, and later, ripening disruption, surface lesions and fungal infestation^3,14,34,35^.

Because of the functional specialization of fruit tissues, and, the many processes that constitute PCI, the progression of this disorder would be expected to also differ across tissues. Therefore, the aim of this work was to examine the components of the molecular, biophysical, biochemical and physiological processes affected by PCI in both the pericarp and columella over short and long-term cold-storage. This could allow us to build a more holistic and integrated view of this phenomenon.

## Results and Discussion

### Respiration and ethylene evolution rates

Carbon dioxide and ethylene production are standard biomarkers for PCI in tomato fruit^2,36^. Respiration supplies the cell with energy^37^, and ethylene is a ubiquitous plant hormone involved in stress response, senescence and fruit ripening^38,39^. In climacteric fruit, the rates of respiration and ethylene production increase with the onset of ripening^1^.

In this study, 2.5 °C, 5 °C and 12.5 °C were used as chilling temperatures, with 2.5 °C and 5 °C expected to induce PCI while 12.5 °C should not and acts as a cold-storage control. The production of these gases was suppressed during the period of chilling and increased up to 100% relative to the control (*p* < 0.05) after rewarming to 20 °C (Supplementary Fig. S1a), which was proportional to the occurrence of PCI. Ethylene production behaved similarly under chilling, but unlike respiration where there was a characteristic burst of CO_2_ within 1 day of rewarming^40^, the occurrence of the ethylene peak was delayed (Supplementary Fig. S1b). This was consistent with a previous study where breaker fruit stored at 3 °C for 3 weeks displayed a peak in this gas after 3 days at 20 °C^41^.

### Chilling Injury Index (CII) and objective color

**Figure 1:**
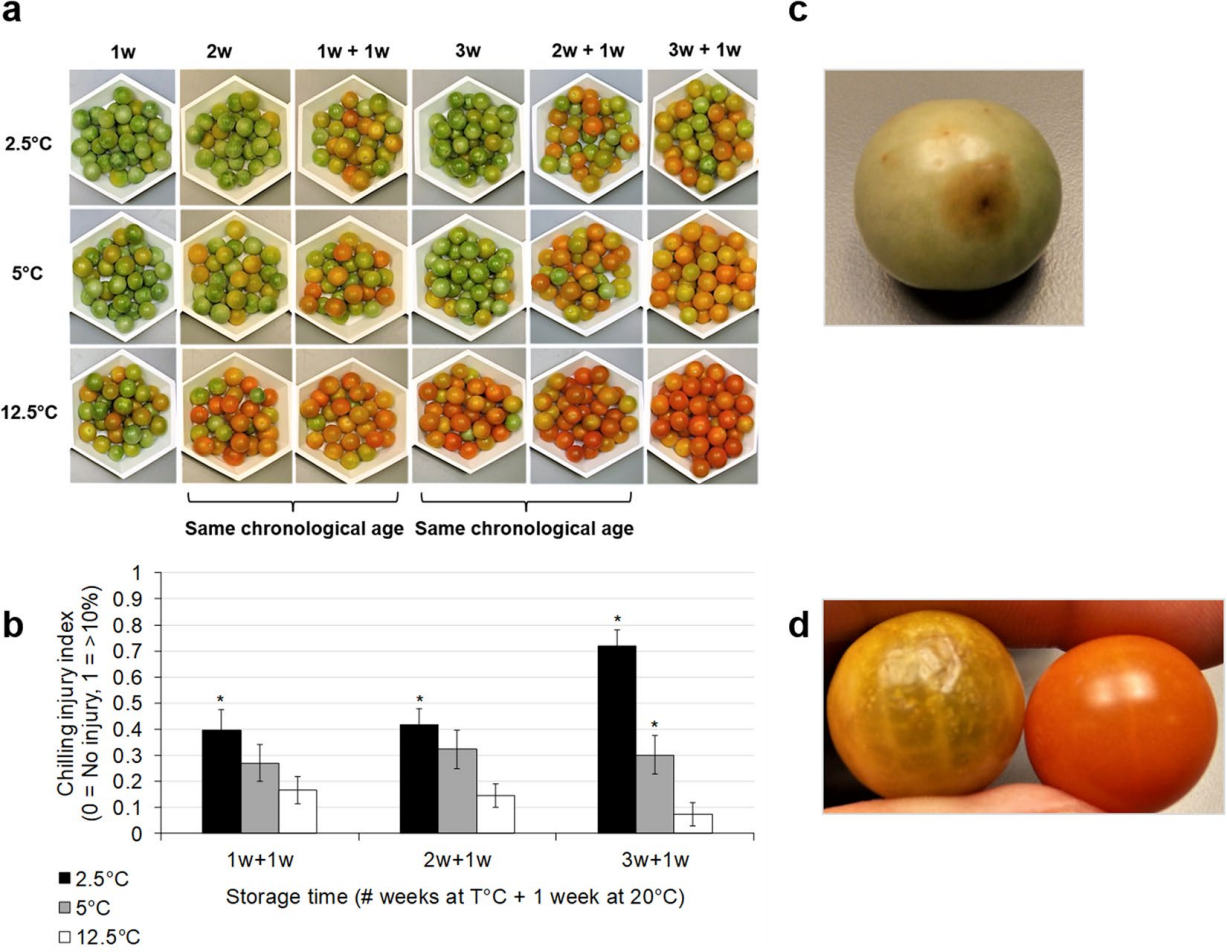
External changes in breaker cherry tomato fruit cv. Sungold after storage. (**a**) Effect of temperature and storage time. Fruit were kept at 2.5, 5 or 12.5 °C for 3 weeks. After 1 (‘1w + 1w’), 2 (‘2w + 1w’) and 3 (‘3w + 1w’) weeks fruit were transferred to 20 °C for 1 week. (**b**) Chilling injury index (mean ± SE). Each column represents the average of 32 fruit per treatment. Columns with asterisks are significantly different (*p* < 0.05) compared to the control (12.5 °C) at a given time point by Kruskal-Wallis test. (**c**) Image of fruit showing decay. Fruit were stored at 2.5 °C for 3 weeks followed by 1 week at 20 °C. (**d**) Images of fruit showing surface pitting. Fruit stored at 2.5 °C for 3 weeks followed by 1 week at 20 °C showing signs of surface pitting (left) and control with no pitting, in fruit stored at 12.5 °C for 3 weeks followed by 1 week at 20 °C (right).

Chilling often leads to poor fruit color development, surface pitting and decay after rewarming, the extent of which is proportional to the severity of the cold stress. The CII encapsulates these data and is expressed as a score from one to four. The rewarmed fruit, especially those stored at 2.5 °C, failed to ripen normally (Fig. 1a), had pitted surfaces (Fig. 1d), and evidence of decay (Fig. 1c), as evidenced by higher CII values, compared to those stored at control temperature (Fig. 1b).

Fruit hue angle values were also assayed, as this is a quantitative and reliable indicator of tomato fruit color changes due to PCI^36^. Hue angle values decreased (15–18%) as redness increased, a trend only seen in control fruit (Supplementary Fig. S2). It confirmed that chilling at both 2.5 and 5 °C adversely affected color development from the first week of cold storage and that rewarming could not reverse these alterations. Chlorophyll degradation, and carotenoid and lycopene accumulation are responsible for red color formation and are inhibited by cold in tomato fruit^42,43^.

### Ion leakage measurements

Structural and conformational changes of cellular membranes are amongst the first physiological events induced by PCI^44^. These alterations reduce membrane selective permeability leading to electrolyte leakage^44,45^. However, we observed no increase in this parameter over time, under 2.5 °C-or 12.5 °C storage, or even when 2.5 °C-stored fruit was rewarmed (Supplementary Fig. S3). At 5 °C, results were not linear since there was a decrease in ion leakage followed by an increase after rewarming (Supplementary Fig. S3). We only recorded ripening-, rather than chilling-induced membrane damage when control fruit was rewarmed. Ion leakage was therefore not an accurate biomarker for PCI in this experiment^35,46^, and our data supports the view that it is highly variable and dependent on pre- and postharvest conditions^35^.

### Fruit tissue *D*-values and spatial characterization

**Figure 2:**
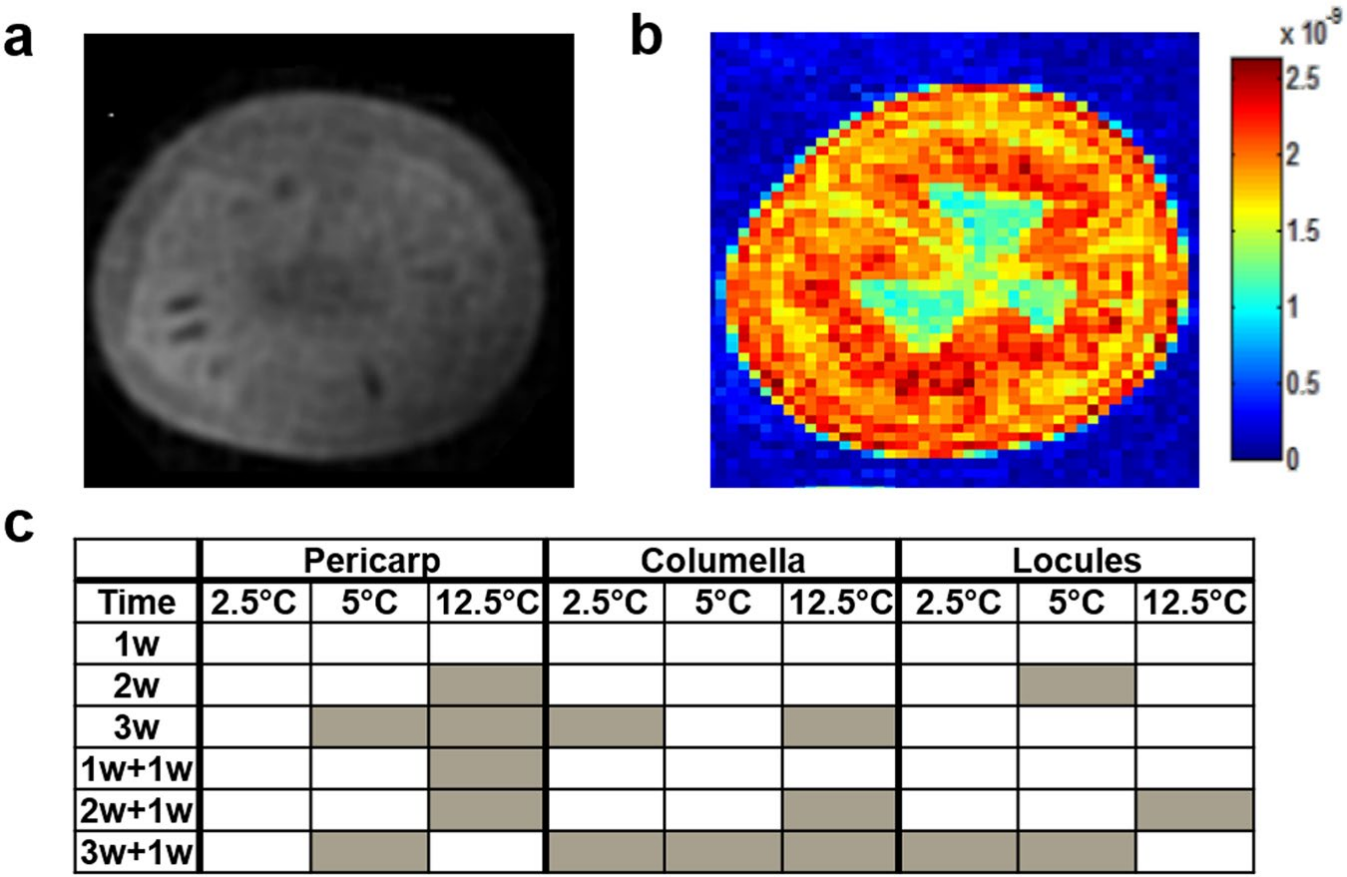
MRI analysis of an equatorial slice of breaker cherry tomato fruit after cold-storage and rewarming. Fruit were kept at 2.5, 5 and 12.5 °C for 3 weeks. After 1 (‘1w + 1w’), 2 (‘2w + 1w’) and 3 (‘3w + 1w’) weeks fruit were transferred to 20 °C for 1 week. (**a**) MRI scan. (**b**) Apparent diffusion coefficient map of an equatorial slice of cherry tomato fruit. The color scale is shown in the color bar. Voxels in red and blue have high and low *D*-values, respectively. (**c**) Schematic representation of *D*-values measured in three fruit tissues. Values within each tissue and temperature were compared to the control which were freshly harvested fruit. Each cell represents 4 replicates, each of them containing 3 fruit. Grey color cells indicate significant differences (*p* < 0.05) and white cells indicate non-significant differences (*p* ≥ 0.05) by Dunnett’s test.

Magnetic resonance imaging (MRI) is a valuable technique for the non-invasive monitoring of tissue physiological status^47^. Diffusion weighted MRI allows the mapping of intra-tissue water mobility, in the form of apparent diffusion coefficient (ADC) images (Fig. 2a,b). ADC (measured as *D*-values) are hypothesized to change due to PCI from chilling-induced biochemical and physiological alterations such as membrane leakage^48^. Non-invasive and simultaneous assessment of the changes in water mobility patterns in the columella, locules and pericarp under chilling and control conditions would be valuable in developing a holistic view of the development of PCI.

Overall, the MRI data illustrated that chilling silences key physiological processes that occur during ripening (Fig. 2c), and that the pericarp, columella and the locules could be clearly resolved based on their water mobility profiles in response to temperature. The pericarp remained unresponsive for the duration of the experiment under 2.5 °C, including rewarming, while the columella and locules were more variable. The locular tissue was the least dynamic fraction under both chilling and control temperatures, and the columella was more responsive after 3 weeks of storage and after rewarming (Fig. 2c).

*D*-values increased mostly, and to a greater magnitude in control fruit, and in chilled fruit after rewarming (Supplementary Fig. S4), which correlates with our ion leakage data (Supplementary Fig. S3). Ripening-associated liquefaction after the transfer to room temperature likely contributed to increased water mobility. Likewise, the only changes in chilled fruit were recorded after prolonged storage (>1 week) or after rewarming (Fig. 2c). This could be attributed to chilling-induced damage since fruit were ripening-inhibited (Fig. 1a) and started to manifest PCI symptoms as revealed by the CII data (Fig. 1b). Cumulative chilling injury likely compromised the tissue’s capacity to undergo normal ripening.

Interestingly, both the ion leakage and ADC data illustrate phenomena associated with water mobility and membrane permeability. While both parameters varied under rewarming after control storage, only ADC appears to be chilling-responsive.

The MRI highlights the need to examine each tissue to characterize PCI’s progression and symptomatology, since the most studied fraction, the pericarp, may not reflect processes occurring in the whole fruit.

**Figure 3:**
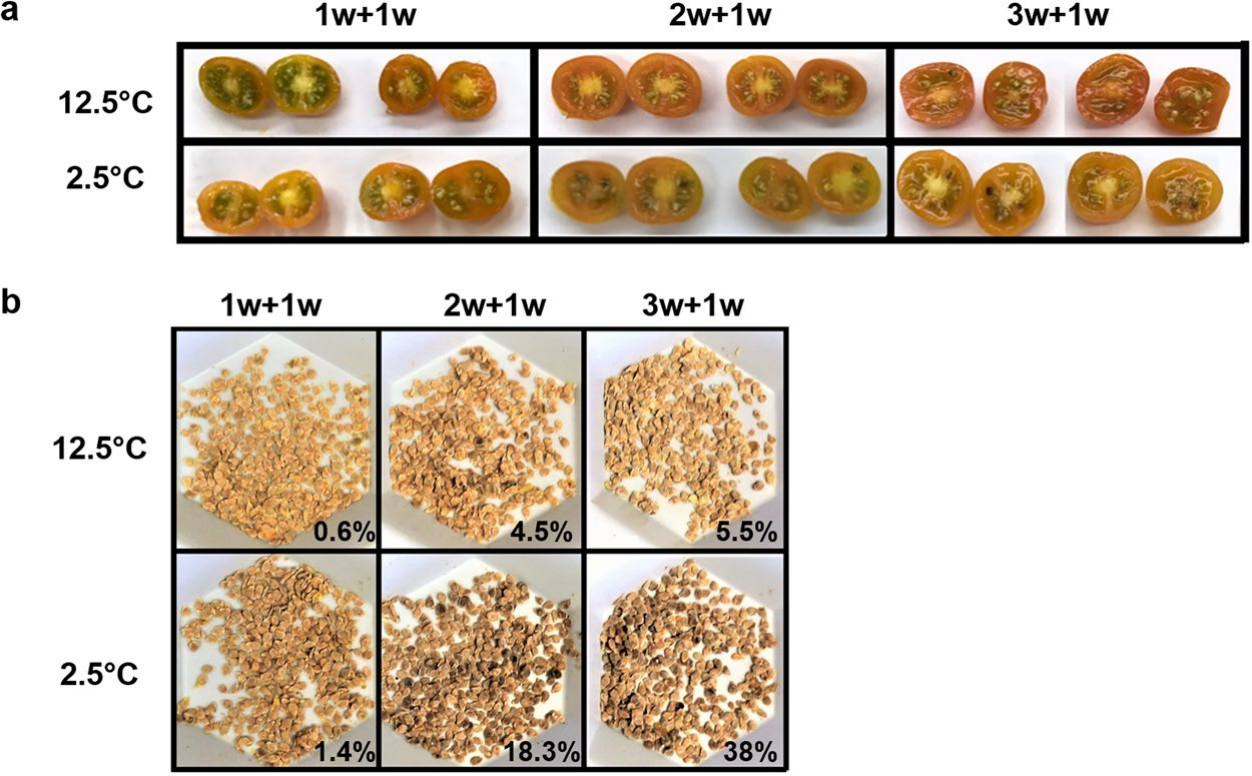
Internal changes of cherry tomato fruit after cold storage and rewarming. Fruit were stored for either 1 (‘1w + 1w’), 2 (‘2w + 1w’) or 3 (‘3w + 1w’) weeks followed by 1 week rewarming. (**a**) Cross-section of stored fruit. (**b**) Seeds extracted from stored fruit and percentages of seeds showing signs of browning and discoloration.

MRI showed that the inner fruit tissues, although traditionally not well-studied, undergo chilling injury. This was further underscored by the higher incidence of seed browning in chilled fruit compared to control fruit (*p* < 0.0001 by Kruskal-Wallis test), after rewarming (Fig. 3a,b). PCI-induced seed browning was also shown in eggplant^49,50^ and pepper^51,52^. Browning is due to the production of melatonin from chilling-induced increases in polyphenol oxidase activity (PPO), its phenolic substrate^53^, and importantly, membrane decompartmentalization, which facilitates PPO access to phenols and the production of brown pigments^54^.

### Malondialdehyde (MDA) content

Cold induces lipid peroxidation of cellular membranes, with MDA as a byproduct^55^. MDA production is considered a biomarker for PCI-induced loss of membrane integrity in tomato fruit both during cold-storage^56,57^, and after rewarming^58,59^.

**Figure 4:**
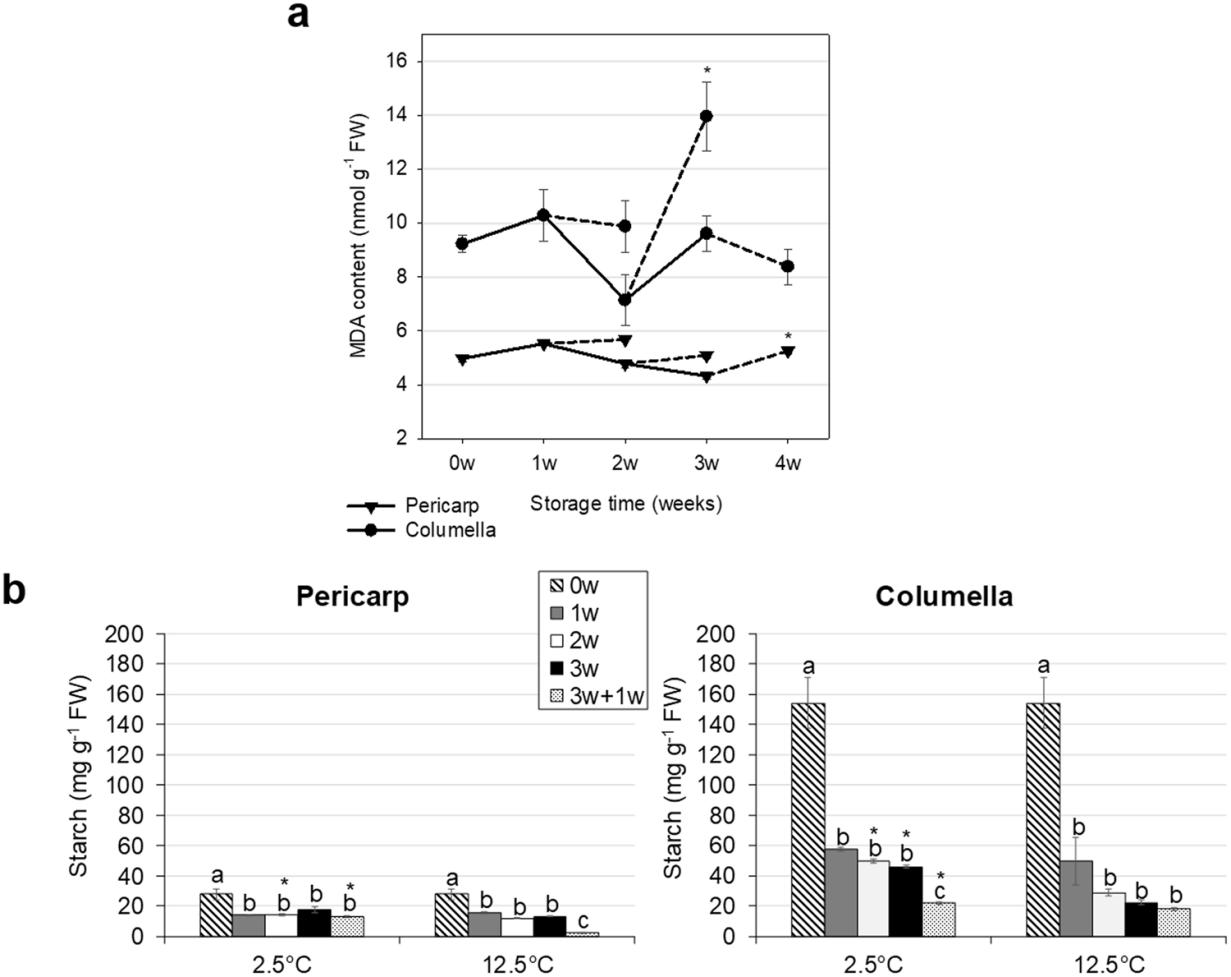
Malondialdehyde (MDA) and starch contents of stored cherry tomato fruit. (**a**) MDA content (mean ± SE) of fruit stored at 2.5 °C, over 3 weeks. After each week of storage, fruit were transferred to 20 °C for 1 week (dashed lines). Each symbol represents the average of 8 fruit per treatment. Asterisks indicate significant differences (*p* < 0.05) between cold storage and the same time point followed by rewarming for 1 week by unpaired *t*-test. (**b**) Starch content (mean ± SE) of fruit stored at 2.5 °C or 12.5 °C up to 3 weeks. After 3 weeks fruit were transferred to 20 °C for 1 week (‘3w + 1w’). Each column represents the average of 6 fruit per treatment. Different letters indicate significant differences (*p* < 0.05) between time points at each tissue and temperature by Tukey’s test. Asterisks indicate differences (*p* < 0.05) between 2.5 °C and 12.5 °C at the same time point by unpaired *t*-test.

MDA production in the pericarp and columella was significantly different (*p* < 0.05) across time points (Fig. 4a), with the greatest changes occurring after rewarming preceded by at least 2 weeks of chilling. MDA content peaked earlier in the columella (2 weeks at 2.5 °C plus rewarming) compared to the pericarp (3 weeks at 2.5 °C plus rewarming) (Fig. 4a). These are signs of a differential response to oxidative stress in both fractions, and that MDA content of the external and internal fruit tissues followed independent programs.

*D-*values and MDA contents report phenomena related to changes in membrane integrity due to PCI, but from different perspectives. *D-*values indicate tissue water mobility status that might result from membrane disruption, while MDA indicates oxidative degradation of the membrane. Interestingly, these parameters showed opposite trends: the pericarp was cold-unresponsive for *D*-values at 2.5 °C (Fig. 2c) but was variable in terms of MDA production. Conversely, the columella fluctuated more in terms of *D*-values under cold, but for MDA production it changed less than the pericarp (Fig. 4a).

### Starch content

Starch is the primary storage compound in green tomato fruit^60^. During postharvest storage of breaker fruit, sugar accumulation will depend on starch degradation, and exogenous factors affecting its breakdown will influence quality. Starch content was higher (41–85%) in columella compared to the pericarp across experimental conditions (Fig. 4b), in agreement with previous studies^61–65^.

Interestingly, both chilling and ripening reduced starch content. Ripening caused more drastic changes, however, starch degradation was still active under cold. This contrasts with the trend seen in other metabolic parameters, i.e., respiration and ethylene production (Supplementary Fig. S1), which were suppressed during chilling. Starch decline was largest after the first week of chilled storage, with a 48.8% and 62.7% decrease in the pericarp and columella, respectively.

When tissues stored for 3 weeks were contrasted with those stored for the same time, but followed by rewarming, starch in the columella decreased under both control and chilling conditions, but only under control conditions in the pericarp. This suggests that after 3 weeks of cold storage, starch degradation in the pericarp reached an irreversible plateau, similar to reports in banana^66^; while starch breakdown was still responsive to rewarming after chilling in the columella.

Total fruit starch content (columella + pericarp) was negatively correlated with respiration (*r* = −0.6, *p* = 0.001) and ethylene evolution rates (*r* = −0.69, *p* = 0.0002) at 2.5 °C. Sugars produced from starch degradation during the cold likely fueled metabolic processes after rewarming through increased respiration. Starch may therefore be an important biomarker of early postharvest chilling injury.

### Gene expression analysis

Changes in the transcriptional abundance of key genes likely occur in ‘waves’ and are among the earliest triggers of the plant cold response^67,68^. Five genes were selected based on their known connection to ripening, changes in redox balance, and cold response. They included genes involved in ethylene biosynthesis and in cold, dehydration, and oxidative stress responses. Their relative abundance was quantified after 1 and 24 hours chilling to investigate rapid changes during short-term storage, and after 3 weeks to investigate changes after prolonged storage. Correlative analyses of the expression patterns between these genes were performed. Significant correlations (*p* < 0.05) may indicate coordinately regulated processes, and it was of interest to determine if they were altered by cold treatment.

#### Ethylene biosynthesis

Ethylene has roles in climacteric fruit ripening and in plant stress response^38,69^. In tomato, the products of *1-aminocyclopropane-1-carboxylic acid* (*ACC*) *synthase* isoform 2 (*ACS2*), and *ACC oxidase* isoform 1 (*ACO1*), encode key enzymes in ethylene biosynthesis. They are both expressed at elevated levels during climacteric and postclimacteric ethylene production^70^.

**Figure 5:**
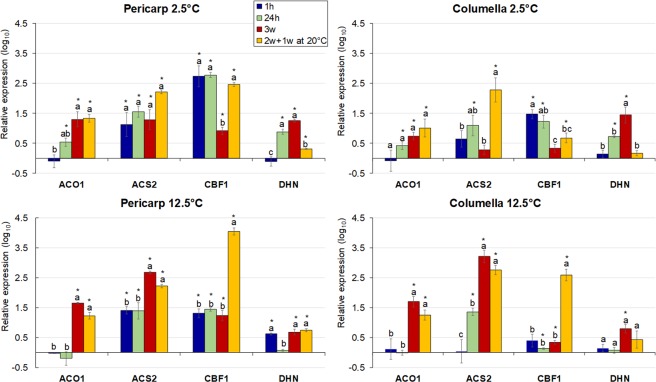
Relative gene expression in the pericarp and columella of cherry tomato fruit. Fruit were stored at 2.5 or 12.5 °C for 1 h, 24 h, 3 weeks, or 2 weeks followed by 1 week at 20 °C. Freshly-harvested breaker fruit were used as the calibrator. Each symbol represents the average of 3 fruit per treatment. Values are the (log_10_) of the mean ± SE. Different letters indicate significant differences (*p* < 0.05) between time points at each tissue and temperature by Tukey’s test. Columns with asterisks are significantly different (*p* < 0.05) compared to the calibrator by unpaired *t*-test.

The expression of *ACO1* and *ACS2* in both pericarp and columella was influenced by the length of storage, but only under control conditions (Fig. 5), consistent with their role in fruit ripening (Supplementary Fig. S1b; Fig. 1a). *ACS2*, was also expressed during post-climacteric ripening in another study^70^. Chilling altered the patterns of gene expression over time. For *ACO1*, transcripts levels were steady in both fractions pre- and post-rewarming, even though ethylene increased 26-fold after rewarming (Supplementary Fig. S1b). Down-regulation of *ACO1* by cold was reported by others^71^, and in our study, was especially apparent in the columella after 3 weeks of cold storage (Fig. 5). *ACS2* expression varied only under conditions that promoted ripening and was more prominent in the columella (Fig. 5).

With *ACO1*, there were no differences in expression between the pericarp and columella fractions under either chilling or control conditions, whereas *ACS2* expression varied spatially under the control temperature. This emphasizes that the expression of both genes followed different spatial dynamics. Both genes were co-expressed at 12.5 °C (Supplementary Fig. S5), however chilling attenuated this strong correlation.

#### Cold stress response

The *C-Repeat Binding Factor 1* (*CBF1*) transcription factor is a master regulator of plant cold stress response^32^, and some studies show that its expression is also induced in tomato fruit during postharvest cold-storage^33,56,72–74^. Unlike *ACS* and *ACO1*, Sl*CBF1* showed a clear spatial differentiation in both chilling and control conditions. Expression was higher in the pericarp compared to the columella (Fig. 5), which suggests that the former might be more responsive to cold stress, possibly due to its external localization.

Under chilling in both tissues, Sl*CBF1* expression peaked at 1 h and was sustained for 24 h in our study, which can be described as an early response (Fig. 5), it then declined to the levels observed at the control temperature, after 3 weeks cold treatment. In other studies, cold storage (temperatures between 2–5 °C) induced expression of Sl*CBF1* for up to 8 h^56^; 8 days^33^, and 14 days^74,75^, however there was no induction at 6 °C in Micro-Tom fruit^76^. The upregulation of Sl*CBF1* may therefore be dependent on fruit developmental stage, the severity of cold stress, and genotype.

After rewarming of the ‘control fruit’, Sl*CBF1* expression increased to levels higher than during chilling (Fig. 5). This may be suggestive of two things: first, that Sl*CBF1* transcripts in chilled tissues were unable to reach the same levels as tissue held in control temperature, since they were developmentally repressed; and second, Sl*CBF1* is involved in ripening independent of *ACO1* or *ACS2*, given the asynchrony of their expression (Supplementary Fig. S5). However, a correlation between endogenous ethylene production and Sl*CBF1* upregulation^72^ could partly explain this behavior and matches our observations (Supplementary Fig. S1b).

#### Dehydration stress response

Dehydrins (DHNs), are protective proteins that accumulate in response to dehydration-associated stresses, including chilling^77^. The expression of the clone FC11CA08-2^76^, here named *DHN*, was analyzed in this study. *DHN* mRNA levels increased only after 24 h and 3w of postharvest chilling in the pericarp, or 3w in the columella (Fig. 5) with decreases in both tissues after rewarming. There were no detectable differences between tissues, however they responded differentially to temperature (Fig. 5). After rewarming, ‘control pericarp’ *DHN* expression was higher than that in the ‘chilled pericarp’, due to ripening taking place in the control. It appears that *DHN* transcript abundance in fruit increases as ripening progresses^78,79^. The magnitude of changes were greater at 2.5 °C compared to 12.5 °C, consistent with a higher requirement for the molecular chaperones encoded under cold-stress^76,80^.

#### Oxidative damage

Prolonged or intense chilling stress induces ROS overproduction, which accelerates cell death^44^. The alternative oxidase pathway (AOX) is activated to minimize ROS levels^81^, and in tomato fruit, *AOX1a* has been associated with enhanced PCI tolerance^82,83^. Therefore, *AOX1a* was studied here. Lipoxygenases (LOXs) catalyze the peroxidation of polyunsaturated fatty acids and are associated with both ripening and redox balance^84^, processes affected by PCI. The expression of the *LoxB* isoform has not been studied during fruit postharvest chilling storage, and was included.

**Figure 6:**
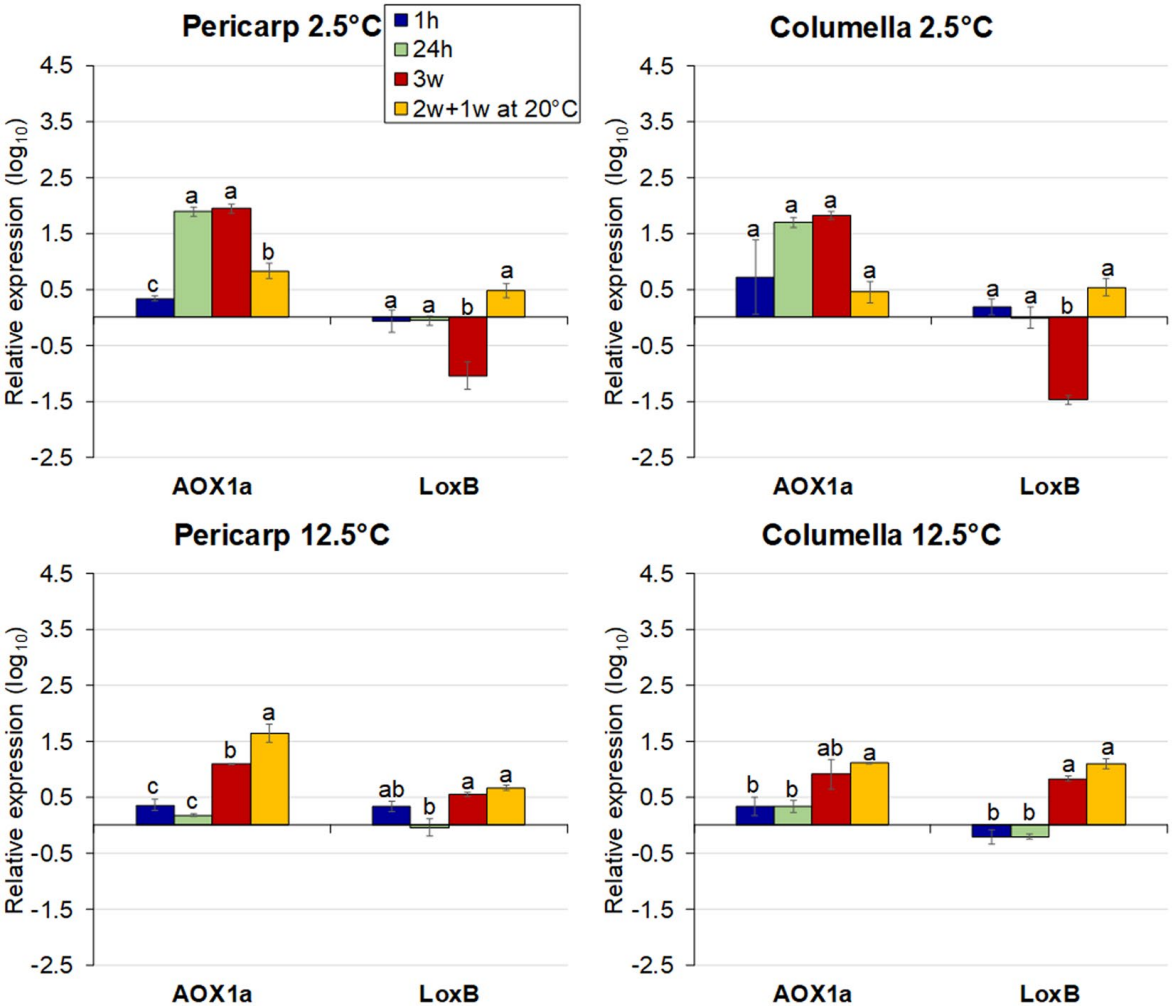
Relative gene expression in the pericarp and columella of cherry tomato fruit. Fruit were stored at 2.5 or 12.5 °C for 1 h, 24 h, 3 weeks, or 2 weeks followed by 1 week at 20 °C. Freshly-harvested breaker fruit were used as the calibrator. Each symbol represents the average of 3 fruit per treatment. Values are the (log_10_) of the mean ± SE. Different letters indicate significant differences (*p* < 0.05) between time points at each tissue and temperature by Tukey’s test. Columns with asterisks are significantly different (*p* < 0.05) compared to the calibrator by unpaired *t*-test.

*AOX1a* expression levels in pericarp and columella were similar, but chilling induced a differential response over time. Transcript levels in the pericarp peaked at 24 h and 3w, (Fig. 6), similar to that seen by Fung *et al*.^82^. The ‘chilled pericarp’ had a reduced *AOX1a* expression after rewarming (Fig. 6) while the ‘chilled columella’ changed little even after rewarming. Under control conditions in both tissues, gradual increases were observed, but rewarming enhanced *AOX1a* expression (Fig. 6), matching ethylene production rates (Supplementary Fig. S1b), consistent with ethylene regulation of this gene^83^. *ACO1*, *ACS2* and *AOX1a*, were co-expressed, but chilling suppressed this correlation (Supplementary Fig. S5). PCI therefore contributes to the uncoupling of ripening-related ethylene biosynthesis, highlighted by the inability of chilled tomato to resume normal ripening after rewarming (Fig. 1a).

*LoxB* expression displayed a mixed spatial-response that varied with temperature. Expression in both pericarp and columella was unchanged at 1 h and 24 h (Fig. 6). After 3 weeks, expression was downregulated, but in contrast, rewarming induced the upregulation of *LoxB* in both tissues. *LoxB* expression matched ethylene production (Supplementary Fig. S1b), consistent with its regulation by this hormone^85,86^. *LoxB* expression also paralleled MDA values after rewarming in the columella (Fig. 4a), in agreement with membrane alterations induced by PCI.

The correlation of *LoxB* with ethylene production rates and ripening was in accordance with the strong correlation between *LoxB* and *ACS2* at 12.5 °C (Supplementary Fig. S5). Interestingly, transcript levels in the ‘control pericarp’ plus rewarming were higher than those of rewarmed tissue after chilling, even though ethylene levels were 1.2-fold higher in the latter. In this case, ethylene production increased in response to chilling-induced stress and not due to ongoing ripening.

### Gene expression correlative patterns

**Figure 7:**
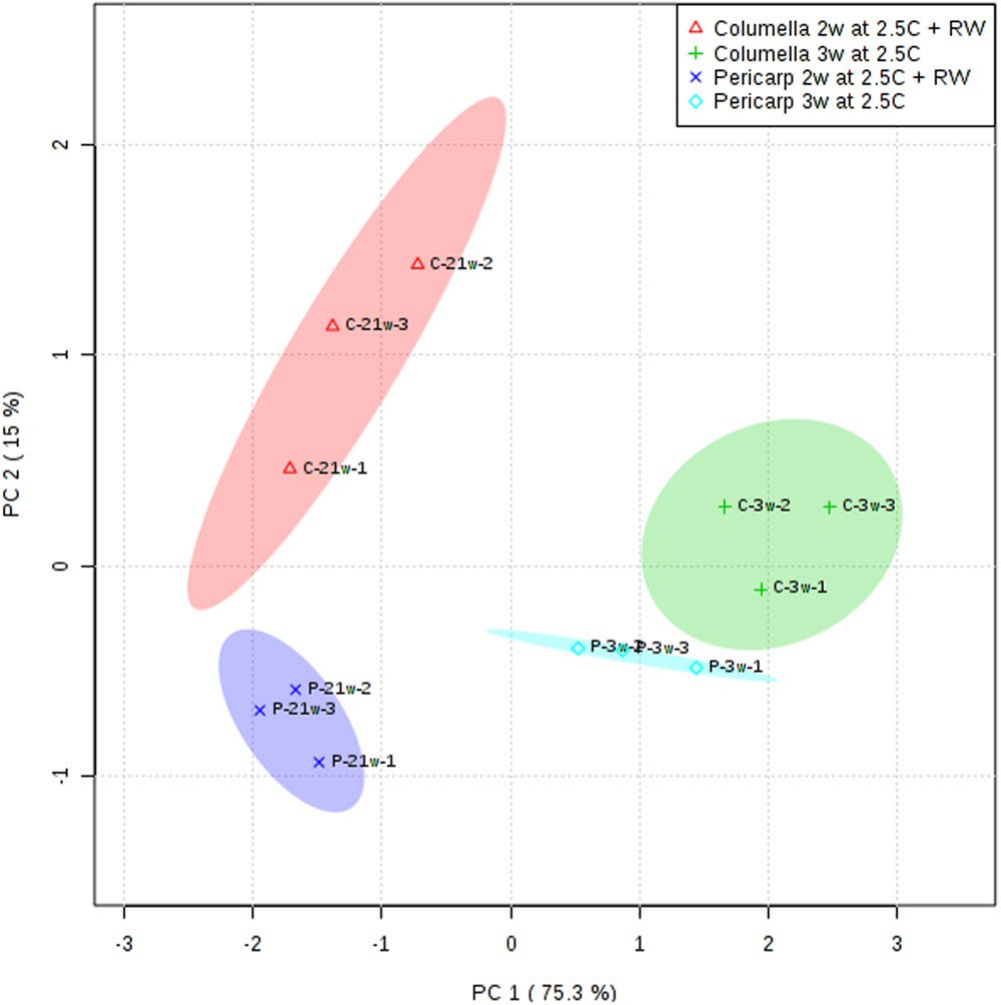
Principal component analysis of the expression of genes in the pericarp and columella of cherry tomato fruit. Data shown here are from fruit kept at 2.5 for 3 weeks or 2 weeks followed by 1 week at 20 °C (‘RW’). Each symbol represents the relative expression values of all genes analyzed per sample, reduced to the first and second principal components. Equal symbols represent biological replicates for the same tissue and time point.

Principal Component Analysis (PCA) was performed to explore the structure of the gene expression data from a spatial perspective with respect to cold storage and rewarming of chilled tissue (Fig. 7). The first and second principal components explained 75 and 15% of the variation present in the data, respectively. Data for the pericarp and columella portions under chilling for 3 weeks separated from the rewarmed tissues. More importantly, the data distinguished among tissues, with the pericarp and columella showing a clear separation even though gene expression differences between cold and rewarming were a greater determinant of the patterns seen on the PCA. Overall this analysis supports the hypothesis of a spatial and temporal differentiation in response to chilling stress at the gene expression level.

## Conclusion

**Figure 8:**
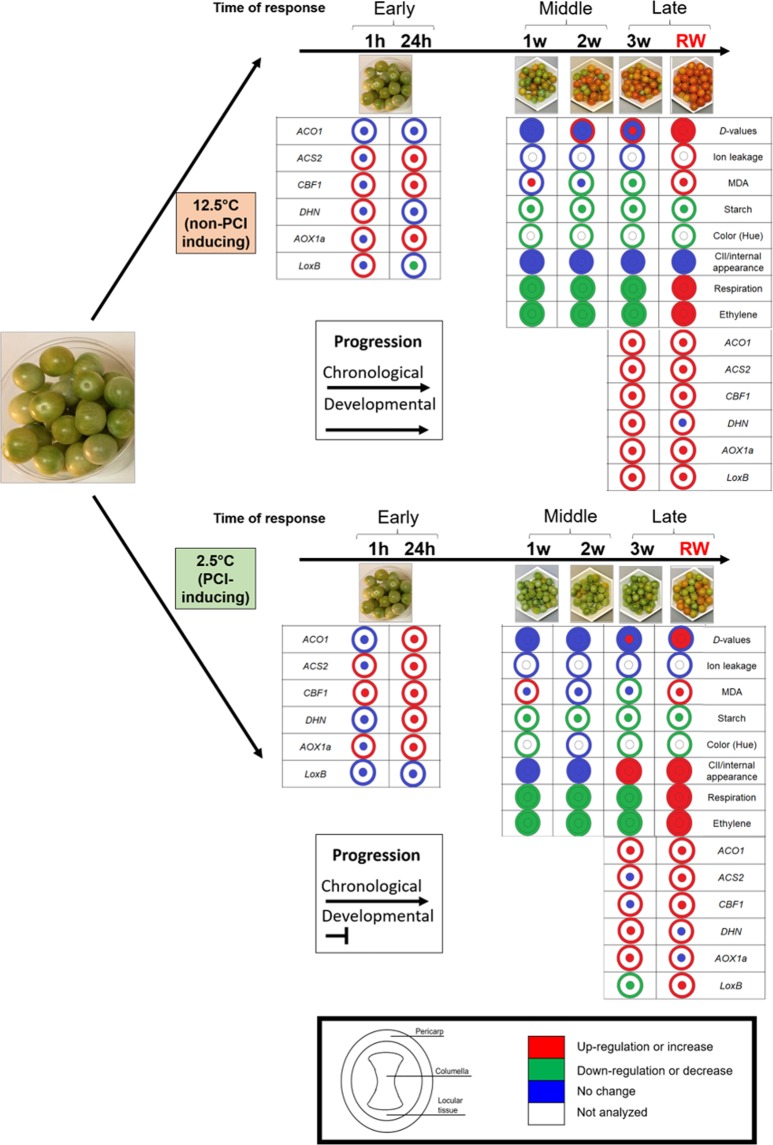
Schematic summary of the effect of storage temperature on the responses of parameters measured in the pericarp, columella or locular tissues of cherry tomato fruit. Fruit were stored at 12.5 °C or 2.5 °C, up to 3 weeks, or followed by storage at 20 °C (rewarming, ‘RW’). Trends of data (increase, decrease or no change), rather than magnitude changes are depicted, and were determined using freshly harvested fruit as the control. Fruit stored at 12.5 °C were compared against harvested breaker fruit so that the data reflected both chronologically and developmental differences. Except for CII and internal appearance, there were no PCI symptoms observed, therefore all other significant changes were related to ripening. PCI disrupts the normal progression of ripening. Comparing cold-stored (2.5 °C) fruit at each time point against harvested breaker fruit, informs on changes between fruit that are also chronologically different, but developmentally closer since cold suppresses maturation. In contrast to fruit at 12.5 °C, the array of changes associated with ripening was not obvious, and in addition, there were cold-injury responses. For the parameters where different tissues were analyzed, the manifestation of these traits could be described as contrapuntal, evidenced as heterogeneity and the decoupling of their response to cold compared to the control.

Postharvest chilling injury (PCI) is a complex multifactorial disorder with detrimental effects on tomato fruit quality and shelf-life. With the aim of representing the tomato fruit as a multilayered and integrated system of response to cold stress, we analyzed PCI impact on different fruit tissues and correlated it with known physiological parameters (Fig. 8).

Overall, cold stress uncoupled key molecular, biochemical, and physiological processes occurring during the normal progression of storage and ripening. Increased water mobility and tissue liquefaction were also disrupted as evidenced by MRI-obtained *D*-values from the pericarp, columella and locular portions, and ion leakage obtained from the pericarp. MRI and color development confirmed three concepts: first, the system’s inability to restore or repair the chilling-affected mechanisms; second, that PCI is cumulative and progressive over time; and third, the need to examine each tissue to characterize PCI’s progression and symptomatology, since the most studied fraction, the pericarp, may not reflect processes occurring in other tissues. Reduced starch breakdown in columella and seed discoloration during cold storage reflect that, besides external changes, PCI extends to internal tissues.

Tissues exhibited heterogeneous patterns of response to PCI at the biophysical, biochemical and molecular levels. *D*-values were intrinsically different in the three tissues under study, and their time evolution and temperature responses were also mixed. Responses to oxidative damage represented by the lipid peroxidation byproduct MDA varied in response to temperature but peaked after rewarming, which again highlights that after crossing a threshold of cumulative cold damage, rewarming aggravates PCI’s manifestation instead of alleviating it. Starch accumulation also showed significant spatial differences, suggesting that tissues may display a sharper specialization at the metabolite than at other levels.

Responses to cold from the perspective of gene expression were highly dependent on the tissue-type, temperature and time of storage, but overall, they paralleled ethylene production trends via stress response or ripening. Some genes seemed to act concertedly across experimental conditions (*ACS2* and *LoxB*), others acted coordinately under either cold or control conditions (*ACO1*, *AOX1a* and *LoxB, CBF1* and *AOX1a*) or under apparently independent programs (*ACO1* or *ACS2* and *CBF1*). Transcript accumulation was (a) higher in the pericarp across conditions (*CBF1*), (b) equally expressed in both tissues (*ACO1*, *AOX1a*), or, (c) dependent on temperature and storage time (*ACS2*, *LoxB* and *DHN*).

Taken together, this evidence reveals the dynamism of cold-stress in the tomato system and suggests that fruit may display specialized mechanisms to elaborate a response to this environmental challenge. It also unfolds numerous questions about the nature of such varied responses among fruit tissues: are they advantageous to the fruit under stress? What is the source of these differences? Would such relationships differ in the fruit from cold-tolerant tomato species? Exploring these questions in a comprehensive way may deepen our knowledge of this complex phenomenon to elaborate long-term, robust solutions.

## Materials and Methods

### Fruit sampling and experimental setup

Cherry tomato (*Solanum lycopersicum* L. var *cerasiforme* cv. Sungold) fruit were harvested at breaker stage^87^. Fruit was obtained from Capay Organic Farm (23804 State Highway 16 Capay, California 95607; 38.707120, −122.070584). After sorting, unblemished and uniform-sized fruit (~2 cm diameter) were washed in a 1:20 dilution of 5% (v/v) sodium hypochlorite and dried in a laminar flow hood. Fruit were left at room temperature for 24 h to and then set at 12.5 °C for 24 h until placed on trays, wrapped with black polyethylene bags and transferred to 2.5, 5 (both PCI-inducing conditions) and 12.5 °C (non-PCI-inducing, ‘control’) for 3 weeks. After 1 week, one fourth of the fruit were transferred to 20 °C for an additional week. This operation was repeated after 2 and 3 weeks of storage at each temperature. For gene expression analysis, fruit were stored at either 2.5 or 12.5 °C for 1 h, 24 h, 3 weeks, or 2 weeks followed by an additional week at 20 °C.

### Gas analysis (Respiration and ethylene evolution rates)

Fruit were placed in 450 mL-jars connected to a humidified air stream (~95% Relative Humidity). A manifold using capillary tubes as flow meters was used to control flow rates. Six biological replicates, each one containing 30 fruit were used for each temperature. Carbon dioxide concentration was measured by taking 1 mL gas samples from a sample port on the sealed containers and injecting into an infrared analyzer for CO_2_. A standard of 0.5136% (v/v) CO_2_ was used for calibration and the difference between inlet and outlet carbon dioxide concentrations was used for calculation of the respiration rates. Ethylene concentration was measured by taking 3 mL gas samples from a sample port on the sealed containers and injecting into a gas chromatograph. A standard of 1.022 ppm ethylene was used for calibration. Both gases were measured daily.

### Chilling injury index (CII)

Fruit were removed from cold rooms and evaluated for CII based on a five-point scale consisting of 3 parameters: surface pitting, uneven ripening and color development, and decay. The severity of the symptoms was assessed visually according to Vega-García *et al*.^88^: 0 = no injury; 1 = <10%; 2 = 11 to 25%; 3 = 26–40%, and 4 = >40%. CII was calculated by using the formula: CII = (Injury level of surface pitting + injury level of uneven ripening + injury level of decay)/3^88^. For each time point and condition, 32 fruit were evaluated individually, and the CIIs were averaged.

### Objective color

A colorimeter with a 2° observer and standard illuminant C was used. Measurements were made in a three-dimensional color space using L* a* b* scale^89^. The Hue angle was calculated as tan−1 (b*/a*)^90^. For each time point and condition, readings were taken from the equatorial region of 12 fruit.

### Ion leakage

Two square pericarp portions (1 cm^2^) from the equatorial region of a fruit were cut and washed in deionized water for 1 minute, blotted dry and placed in a 50-mL tube with 0.2 M D-mannitol. Tubes were shaken for 1 hour at room temperature and the initial conductivity was measured, followed by freezing the tubes at −20 °C for 2 days. Samples were thawed at room temperature for 24 hours, shaken for 1 hour and total conductivity was measured. Ion leakage was quantified as the ratio between initial conductivity and total conductivity multiplied by 100^91^. Four biological replicates were used, each consisting of a single pericarp portion from 5 fruit.

### Magnetic resonance imaging (MRI)

Fruit were removed from controlled-temperature rooms at least 3 hours before analysis. The apparent diffusion coefficient (ADC) of water was measured by diffusion weighted MRI on an NMR spectrometer as previously reported, and expressed as *D*-values^48^. A total of 4 replicates were used, each one containing 3 fruit. Each replicate was placed in a circular holder and introduced into the equipment for reading.

### Malondialdehyde (MDA) determination

The protocols of Hodges *et al*. (1999) and Nagababu *et al*. (2010), were modified in this assay^92,93^. The pericarp and columella were excised and homogenized in 2 volumes of cold 20% (w/v) trichloroacetic acid (TCA) solution, followed by centrifugation at 12,000 × *g* for 20 min. Three hundred microliters of supernatant were mixed with 300 µL of 0.67% (w/v) thiobarbituric acid (TBA). The solution was incubated at 95 °C in a water bath for 25 min, cooled in an ice bath for 15 min and then centrifuged at 12,000 × *g* for 10 min. The absorbance (532 nm) was measured spectrophotometrically by taking 400 µL of the mixture, and the value for non-specific absorption at 600 nm was subtracted. MDA content was quantified by using a standard curve in concentrations of 0 to 6 nmol of malonaldehyde bis(dimethyl acetal), followed by reaction with TBA as described above. Working standards were prepared fresh daily. Eight biological replicates were used, each consisting of tissue excised from a single fruit.

### Starch Assay

The protocol of Smith and Zeeman (2006) was modified in this assay^94^. The pericarp and columella were boiled three times in 5 mL of 80% (v/v) ethanol each time, discarding the ethanol. The tissue was homogenized to a powder and resuspended in 5 mL distilled water. Three aliquots (500 µL each) of the homogenate were autoclaved for 45 min, and 500 µL 200 mM sodium acetate pH 5.5 was added to each. To two aliquots, six units of α-amyloglucosidase and 0.5 units of α-amylase were added, and all samples were incubated at 37 °C overnight to digest starch into glucose. Reducing sugars were assayed using freshly-made 3,5-dinitrosalicylic acid (DNS) reagent following the protocol of Dong *et al*.^95^. The concentration of the reducing sugars in the samples was determined from the standard curve, and those in the ‘no-enzyme’ controls were subtracted from those digested with the enzymes. Six biological replicates, each consisting of tissue excised from a single fruit, and three technical replicates were used.

### Quantitative Real-Time PCR (RT-qPCR)

Total RNA was isolated from 100 mg samples from the pericarp and columella^96^ and DNase-treated with the TURBO DNA-free™ Kit (Life Technologies, Carlsbad, CA, USA). cDNA was synthesized from 500 ng of RNA using random primers with the High-Capacity cDNA Reverse Transcription Kit (Applied Biosystems, Foster City, CA, USA). The cDNA was diluted 40-fold and RT-qPCR was performed in a 10 μL reaction as follows: 0.4 μL of nuclease-free water, 0.3 μL of 10 μM forward primer, 0.3 μL of 10 μM reverse primer, 5 μL of iQ™ SYBR® Green Supermix (Bio-Rad, Hercules, CA, USA) and 4 μL of diluted cDNA as template. The reactions were placed in a real-time thermocycler (CFX96 Touch™ Real-Time PCR Detection System, Bio-Rad, Hercules, CA, USA) using the following parameters: 95 °C for 3 minutes, and 40 cycles of 95 °C for 10 seconds and 60 °C for 30 seconds. Primers were design based on the cDNA sequences published on Sol Genomics (Supplementary Table S1), with size amplicons between 107–181 bp. The reaction efficiency for each pair of primers (Supplementary Table S1) was between 90–107%. The specificity of the assay for each gene was validated through a melt-curve analysis. Relative quantification and normalization were determined by the Pfaffl method^97^. The tomato actin gene (*SlACT7*) was used as a reference and freshly harvested breaker fruit was the calibrator sample. Three biological and three technical replicates were used for each experimental condition. A biological replicate consisted of tissue excised from a single fruit.

### Experimental design and statistical analysis

A complete randomized factorial design was used, with type of tissue, temperature and time of storage as factors, unless otherwise specified.

Data were statistically analyzed using SAS software Version 9.4, RStudio Version 1.1.419 or Microsoft Excel Version 1804; and graphed using SigmaPlot version 12.0, RStudio, Microsoft Excel or Metaboanalyst Version 4.0^98^. The following R packages were used: *corrplot*^99^, *pca3d*^100^ and *factoextra*^101^.

Analysis of variance (ANOVA) or unpaired *t*-test were performed to detect significant differences among and within treatments. For mean comparison, Tukey’s and Dunnett’s tests were used with *α* = 0.05. Principal component analysis (PCA) was performed to analyze gene expression patterns during cold storage and after fruit rewarming. Correlation statistics were carried out using Pearson’s or Spearman’s correlation coefficients. Kruskal-Wallis test was used as a nonparametric alternative to ANOVA. Confidence interval (CI) or standard error (SE) were used as measures of variability of the data.

## Supplementary information

Supplementary Information

## Data Availability

All of the materials, data and associated protocols will be made available upon request without preconditions. All data generated from this work and not presented in the figures are in the Supplemental Information File.
