# Supplementary material for: Integrative analysis of postharvest chilling injury in cherry tomato fruit reveals contrapuntal spatio-temporal responses to ripening and cold stress
Source: Sci Rep. 2019 Feb 26;9:2795. doi: 10.1038/s41598-019-38877-0 (PMC6391400; doi:10.1038/s41598-019-38877-0)
Supplement: Supplementary file 1 — Supplementary Information [file 41598_2019_38877_MOESM1_ESM.docx]

Supplementary Information for
 **Integrative analysis of postharvest chilling injury in cherry tomato fruit reveals contrapuntal spatio-temporal responses to ripening and cold stress**

Karin Albornoz^1^, Marita I. Cantwell^1^, Lu Zhang^2,3^ and Diane M. Beckles^1*^

*Corresponding Author:

Diane M Beckles,

Department of Plant Sciences, Mail Stop 3

University of California

Davis, CA 95616, USA

**List of tables and figures in supplementary information**

Supplementary Table S1. Primers used for real-time quantitative PCR.

Supplementary Figure S1. Gas evolution rates of cherry tomato fruit.

Supplementary Figure S2. Hue angle of chilled cherry tomato fruit.

Supplementary Figure S3. Ion leakage of chilled cherry tomato.

Supplementary Figure S4. Apparent diffusion coefficient of water of cherry tomato.

Supplementary Figure S5. Correlograms of correlation matrices of genes of cherry tomato.

**Supplementary Table S1. Primers used for real-time quantitative PCR.**

| **Gene ID** | **Name** | **Nucleotide sequence (5'- 3')** | **Amplicon size (bp)** |
| --- | --- | --- | --- |
| Solyc03g026280 | CBF1 Fwd | AGGGGAATCAGGAAGAGGAA | 156 |
|  | CBF1 Rev | ACAAGCAGAACGGCCTCTTA |  |
| Solyc04g082200.2 | DHN (FC11CA08) Fwd | TCACAAGGAAGAATCGAAAGC | 138 |
|  | DHN (FC11CA08) Rev | GACACCACCTCCGTCTTGTTATG |  |
| Solyc07g049530.2 | ACO1 Fwd | ACAAACAGACGGGACACGAA | 181 |
|  | ACO1 Rev | CTCTTTGGCTTGAAACTTGA |  |
| Solyc01g095080.2 | ACS2 Fwd | GAAAGAGTTGTTATGGCTGGTG | 107 |
|  | ACS2 Rev | GCTGGGTAGTATGGTGAAGGT |  |
| Solyc03g078400.2 | ACT7 Fwd | GCTATCCAGGCTGTGCTTTC | 157 |
|  | ACT7 Rev | CAGTAAGGTCACGACCAGCA |  |
| Solyc01g099190 | LoxB Fwd | CACATCCAGACGGAGATCAA | 119 |
|  | LoxB Rev | GCCCATGTCATTCACTGCTA |  |
| Solyc08g075540.4 | AOX1a Fwd | CTTCGTTTCCCCACTGATGT | 156 |
|  | AOX1a Rev | AGCTTTGATCCATCCACCAC |  |


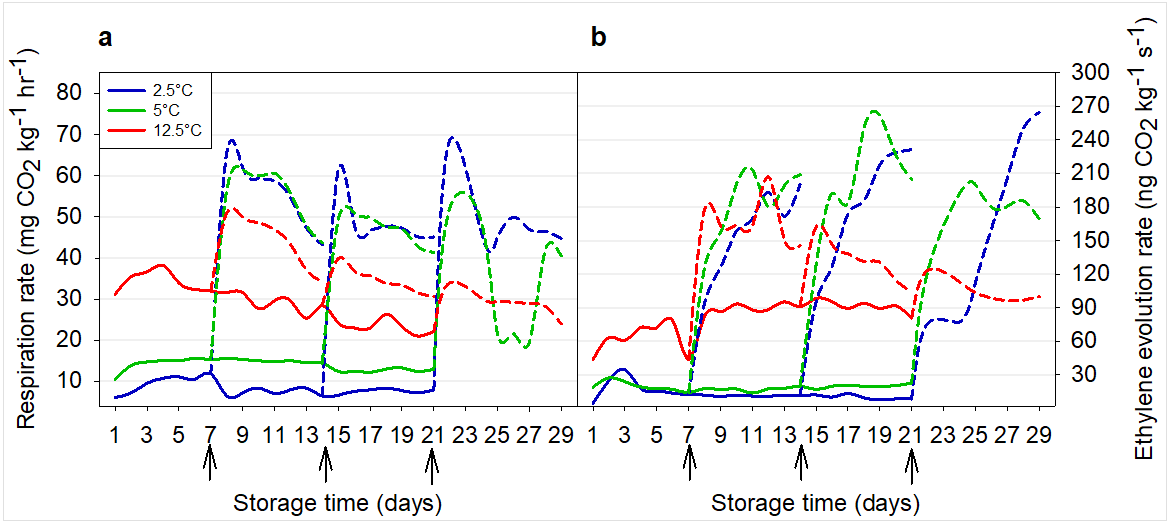


**Supplementary Figure S1. Gas evolution rates (means) of cherry tomato fruit. Arrows indicate the transfer of fruit after 7, 14 and 21 days at 2.5, 5 or 12.5°C, to rewarming conditions (20°C) for 1 week (dashed lines). (a) Respiration rates. (b) Ethylene evolution rates. Each point represents the average of 6 replications per treatment. Each replication consisted of 30 tomato fruit.**

**
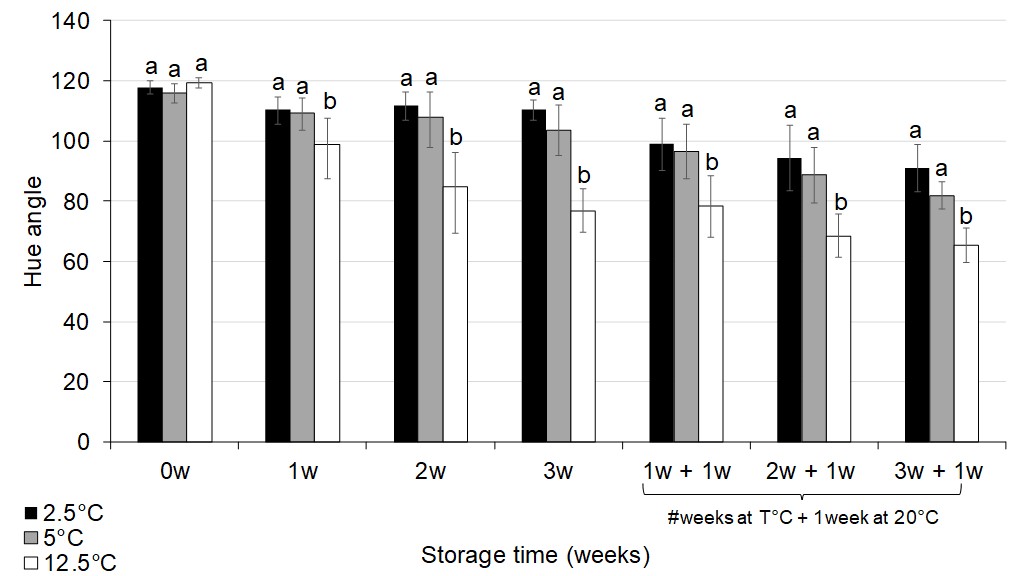
**

**Supplementary Figure S2. Hue angle (mean ± 95% CI) of cherry tomato fruit. Fruit were stored at 2.5, 5 and 12.5°C for 3 weeks. After 1, 2 and 3 weeks fruit were transferred to 20°C for 1 week. Each column represents the average of 12 fruit per treatment. Bars with different letters are significantly different (*p* < 0.05) at a given time point by Tukey’s test.**


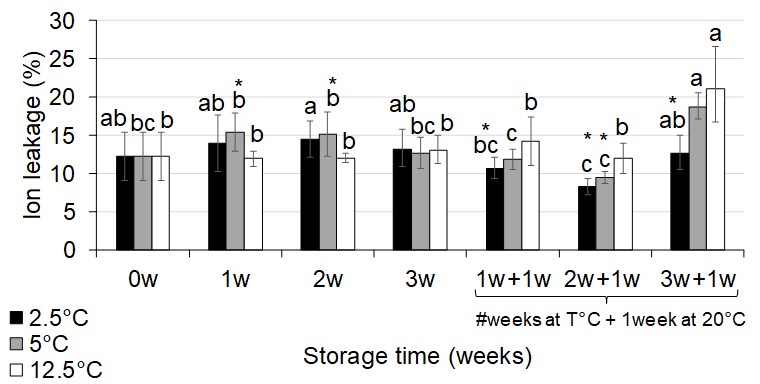


**Supplementary Figure S3. Ion leakage of cherry tomato fruit. Percentage of ion leakage (mean ± 95%CI) of fruit stored at 2.5, 5 or 12.5°C for 0, 1, 2 and 3 weeks followed by storage at 20°C for 1 additional week. Each column represents the average of 5 fruit per treatment. Different letters indicate significant differences among time points at each temperature. Columns with asterisks are significantly different (*p* < 0.05) from the control (12.5°C) at a given time point by Tukey’s test.**


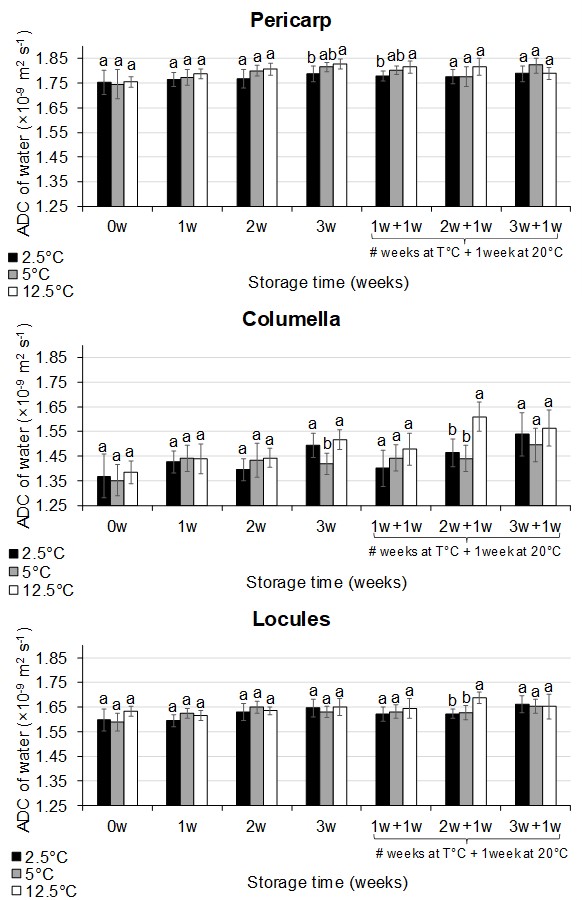


**Supplementary Figure S4. Apparent diffusion coefficient of water (*D*-value mean ± 95% CI) of three tissues within an equatorial slice of a single cherry tomato fruit. Fruit were stored at 2.5, 5 or 12.5°C for 3 weeks. After 1, 2 and 3 weeks fruit were transferred to 20°C for 1 week. Each column represents the average of 12 fruit per treatment. Bars with different letters are significantly different (*p* < 0.05) for each tissue at a given time point by Tukey’s test.**


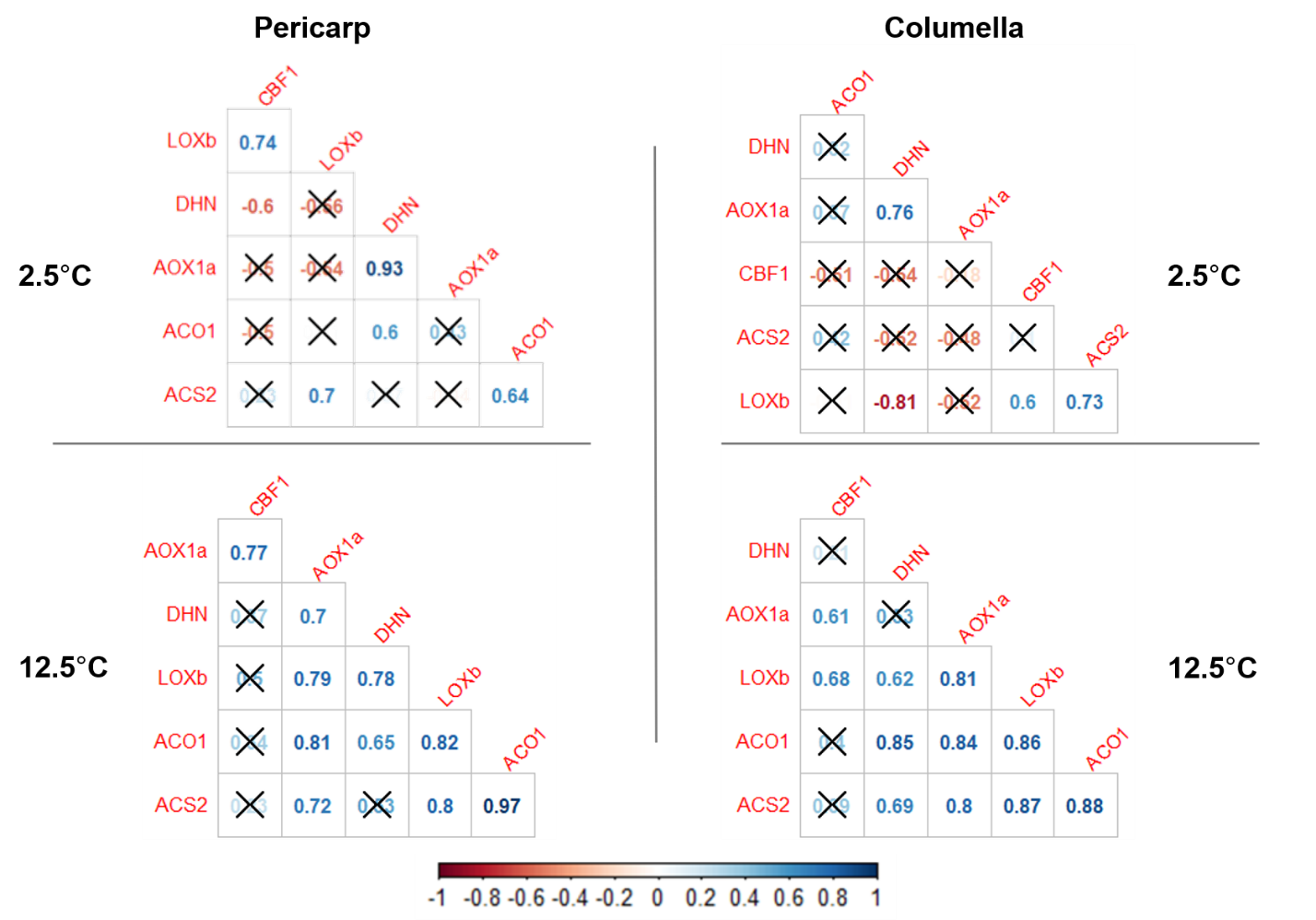


**Supplementary Figure S5. Correlograms of correlation matrices of genes in the pericarp and columella fractions of cherry tomato fruit stored at 2.5 or 12.5°C for 1h, 24h, 3 weeks, or 2 weeks followed by 1 week at 20°C. Values represent the Pearson correlation coefficient from a pair of genes at each temperature and tissue, across time points. Crossed values indicate non-significant correlations (*p* > 0.05).**
